# Evaluation of Cannabidiol Oil’s Effects on Sedation, Behavioral Responses to Handling, and Nociceptive Thresholds in Healthy Cats

**DOI:** 10.3390/ani15131987

**Published:** 2025-07-06

**Authors:** Kannika Wanapinit, Sirirat Niyom, Panisara Suriyawongpongsa, Sakunrat Khathatip, Kaittisak Tancharoen, Sittiruk Roytrakul, Sekkarin Ploypetch

**Affiliations:** 1 Kasetsart University Veterinary Teaching Hospital, Faculty of Veterinary Medicine, Kasetsart University, Bangkok 10900, Thailand; kannika.wana@ku.th (K.W.); sakunrat.kha@ku.th (S.K.); 2 Department of Companion Animal Clinical Sciences, Faculty of Veterinary Medicine, Kasetsart University, Bangkok 10900, Thailand; 3 Veterinary Clinical Studies Program, Faculty of Veterinary Medicine, Graduated School, Kasetsart University, Nakorn Pathom 73140, Thailand; panisara.sur@ku.th; 4 Department of Large Animal and Wildlife Clinical Science, Faculty of Veterinary Medicine, Kasetsart University, Nakhon Pathom 73140, Thailand; fvetkata@ku.ac.th; 5 National Center for Genetic Engineering and Biotechnology, National Science and Technology, Development Agency, Pathum Thani 12120, Thailand; sittiruk@biotec.or.th; 6 Department of Clinical Sciences and Public Health, Faculty of Veterinary Science, Mahidol University, Nakhon Pathom 73170, Thailand; sekkarin.plo@mahidol.ac.th

**Keywords:** behavior, cannabidiol, cat, compliance, nociceptive threshold, sedation

## Abstract

This study explored the effects of cannabidiol (CBD) on clinically relevant parameters, including sedation, compliance, and temperament, as well as mechanical nociceptive thresholds in healthy cats. Nine client-owned cats (3.44 ± 2.35 years, mean ± standard deviation) were assessed prior to (baseline) and 30 min, 1, 2, 4, 8, 12, and 24 h after oral administration of 8 mg/kg CBD oil via capsule. Sedation scores increased significantly 2, 4, and 8 h post administration compared to baseline (all medians = 1 vs. 0 at baseline; *p* < 0.001). Compliance and temperament scores were significantly reduced 2 and 4 h post dosing, with median scores decreasing from 1 at baseline to 0 after 2 and 4 h for both parameters (*p* < 0.001 and *p* = 0.012, respectively). Mechanical nociceptive thresholds and sensitivity, assessed using an algometer and von Frey filaments, respectively, along with physiological parameters (heart rate, respiratory rate, and body temperature), remained unchanged across timepoints. These results indicate that 8 mg/kg CBD induces mild sedation and reduces handling resistance in healthy cats without affecting nociceptive thresholds or physiological stability. Therefore, CBD may facilitate non-painful procedures requiring animal cooperation; further controlled studies are warranted to confirm these findings.

## Simple Summary

This study aimed to provide an initial assessment of the effects of cannabidiol (CBD) oil on sedation, compliance during handling, temperament, and mechanical nociceptive thresholds in healthy cats. Nine adult cats were administered 8 mg/kg of CBD, and their responses were monitored over a 24 h period. The results revealed a mild but significant sedative effect, with increased sedation scores 2, 4, and 8 h post administration. Compliance and temperament scores were reduced 2 and 4 h post administration. CBD did not significantly alter mechanical nociceptive thresholds or physiological parameters, including heart rate, respiratory rate, and body temperature. These findings suggest that CBD induces mild sedation and enhances compliance without affecting nociceptive thresholds or physiological stability.

## 1. Introduction

Cannabidiol (CBD), a non-psychoactive compound derived from *Cannabis sativa* and *Cannabis indica*, has garnered considerable attention for its potential therapeutic applications in both human and veterinary medicine [1,2,3]. CBD primarily interacts with the endocannabinoid system (ECS), a complex network of receptors that regulate various physiological processes, including pain perception, emotion, anxiety, stress response, appetite, inflammation, and immune function [4]. Through its interaction with the ECS, CBD is hypothesized to provide benefits in managing a range of medical conditions and promoting physiological balance.

The therapeutic effects of CBD are believed to be mediated by its action on multiple receptors within the ECS, notably cannabinoid receptors 1 and 2 (CB1 and CB2). CB1, predominantly located in the central nervous system, is involved in modulating pain, mood, and memory, while CB2, primarily found in peripheral tissues, plays a role in immune response and inflammation [4,5,6,7]. Beyond these, CBD also acts on non-cannabinoid receptors, such as the serotonin 5-HT_1A_ receptor, which is involved in anxiety, depression, and pain regulation [8,9,10], as well as the transient receptor potential vanilloid 1 (TRPV1) receptor, known to mediate pain sensation [11,12]. These diverse interactions underscore CBD’s potential as an alternative treatment for anxiety, stress, and pain in animals.

Despite promising preclinical data, the use of CBD in small animal care is largely driven by anecdotal reports and owner or practitioner perceptions [2,13,14,15]. Surveys of dog owners and veterinarians indicate growing interest and perceived benefits of CBD products, particularly for managing pain and anxiety [2,13,14]. Nevertheless, rigorous scientific evidence supporting its efficacy in dogs and cats remains limited [16]. Positive outcomes have been reported, such as pain relief in canine osteoarthritis [17,18] and reduced anxiety during caregiver separation in dogs [19] and cats [20]. However, the findings remain inconsistent in their effectiveness. A study using a fireworks model of noise-induced fear in dogs found no significant anxiolytic effect at a CBD dose of 1.4 mg/kg [21]. Similarly, a study on shelter dogs reported that daily administration of CBD oil for 45 days had no significant effect on stress-related behavioral patterns, such as stereotyped behavior and displacing activities [22].

In cats, most existing studies have focused on pharmacokinetics and safety profiles [23,24,25,26], while evidence regarding behavioral effects—particularly in healthy individuals—remains scarce. To date, few studies have systematically examined the potential of CBD to induce sedation or mitigate anxiety-related behaviors, such as resistance to handling or fear-induced aggression. This gap underscores the need to investigate the behavioral effects of CBD in cats. In terms of safety, adverse effects such as lethargy, excessive salivation, and elevated liver enzymes have been reported in cats, particularly at higher or prolonged doses [23,24,25,26,27]. In dogs, prolonged CBD administration over 1–3 months has been associated with elevated alkaline phosphatase levels, suggesting potential impacts on liver function [17,28]. These findings, along with the limited research available, highlight the need for further studies to conclusively determine the safety and efficacy of CBD in small animals.

Given the medicinal potential of CBD, high-throughput technologies such as genomics, transcriptomics, proteomics, lipidomics, and metabolomics have become valuable tools in exploring its multifaceted effects [29]. Among these, proteomics has shown promise in detecting and analyzing proteins potentially associated with cannabinoids like tetrahydrocannabinol (THC) and CBD [30]. However, existing proteomic studies on cannabinoid activity remain limited and primarily focus on specific tissues such as human keratinocytes, astrocytes, or animal brain tissues [31]. In this study, proteomic profiling was included as an exploratory tool to complement behavioral observations and to generate hypotheses for future research on potential protein markers or mechanisms associated with CBD effects in cats.

The present study aimed to explore the effects of CBD on clinically relevant parameters in cats, including sedation, behavioral responses to handling, and nociceptive thresholds. In addition, plasma cannabinoid compounds potentially associated with these effects were analyzed using nanoscale liquid chromatography–tandem mass spectrometry (nanoLC-MS/MS). Assessments were conducted at multiple time points following administration to test the hypothesis that CBD oil induces sedation and reduces resistance and stress during handling, thereby enhancing cooperation during experimental procedures, and it increases mechanical nociceptive thresholds.

## 2. Materials and Methods

### 2.1. Animals

The study protocol was approved by the Institutional Animal Care and Use Committee of Kasetsart University (Approval No. ACKU65-VET-042). Healthy, client-owned cats aged 1 to 7 years were recruited as volunteer participants. Written informed consent was obtained from their owners. Health status was confirmed based on physical examination, medical history, and hematological and biochemical analyses, including complete blood count, blood urea nitrogen (BUN), creatinine, alanine aminotransferase (ALT), aspartate aminotransferase (AST), albumin, and total protein. Cats were excluded if they showed signs of illness, had abnormal blood parameters, any diagnosed medical condition, received medications within one month prior to the study, or had a history of orthopedic or neurological disorders.

Each cat was brought into the study room one day prior to testing. The room was equipped with standard feline amenities, including a litter box, bed, and toys. Commercial dry kibble food was provided twice daily, and water was available ad libitum.

### 2.2. Protocols

The main experiment was conducted in nine healthy client-owned cats, comprising seven males and two females. The cohort included seven domestic shorthair (DSH) cats and two Persian cats, with a mean age of 3.44 ± 2.35 years (mean ± standard deviation (SD)) and a mean weight of 4.87 ± 0.71 kg (mean ± SD). Each cat received a single oral dose of 8 mg/kg CBD oil in a capsule, administered two hours after morning feeding. The CBD product used was a CBD-enriched medical cannabis formulation (CBD: THC ≥ 20:1), containing 100 mg/mL of CBD (Sibannac CBD cannabis oil; GPO, Pathum Thani, Thailand).

The selected dosage was determined based on preliminary data obtained from a dose-finding trial in six healthy cats (four females, two males; five DSH, one Persian), aged 1 to 7 years and weighing 3.8–5.4 kg, in which different doses were evaluated for sedative effects. Cats were randomly assigned to receive CBD oil at 2, 4, or 8 mg/kg (n = 2 per group) via oral capsule. Sedation was assessed using a numerical scoring system adapted from previous feline studies [32,33,34,35] (Table A1) at baseline and 30 min, 1, 2, 4, and 8 h post administration. Minor modifications were made to the scoring system, including the addition of a moving toy as a stimulus alongside gentle stroking and handclapping to assess responsiveness. A single blinded evaluator scored all cats at every timepoint to ensure consistency. Cats receiving 2 or 4 mg/kg of CBD showed no signs of sedation (sedation score = 0 at all timepoints). In contrast, those administered 8 mg/kg exhibited signs of mild sedation, such as staggering while walking, with a sedation score of 1 observed consistently from 1 to 8 h post administration. Based on these findings, the 8 mg/kg dose was selected for use in the main experiment. This dose also aligns with a previous study [24] in which two out of four cats that escalated to a dose of 8.3 mg/kg CBD and 0.31 mg/kg THC exhibited lethargy, whereas lethargy was reported in only one of four cats receiving lower doses (CBD 5.5 mg/kg and THC 0.21 mg/kg, and CBD 2.8 mg/kg and THC 0.1 mg/kg). In addition, no serious adverse effects were reported at any of these doses, suggesting that 8 mg/kg CBD may be well tolerated in feline subjects.

In the main experiment, cats were assessed for sedation level, temperament, and compliance at baseline (prior to CBD administration) and 30 min, 1, 2, 4, 8, 12, and 24 h after receiving 8 mg/kg of CBD. Sedation was evaluated using the same numerical scale employed in the preliminary trial (Table A1). Compliance and temperament were evaluated according to established scoring systems from earlier feline research [36,37]. The compliance score ranged from 0 to 3, where 0 indicated no resistance to handling, 1 indicated minimal resistance to handling, 2 indicated struggling and being difficult to handle, and 3 indicated extreme struggling with or without urination or defecation [36]. The temperament score was modified from a previous study [37] and included the following: 0 = interactive and interested when approached, showing no objection to physiological assessments and blood sampling; 1 = scared or nervous, hiding in a corner when approached, or tolerating assessments but appearing rigid; and 2 = exhibiting aggressive behaviors, such as biting or scratching, which preclude some or all assessments. Minor modifications were made to focus on behavioral responses to physiological assessments and blood sampling, rather than clinical examinations as used in the original study. In that study, temperament was similarly scored on a 0–2 scale: a score of 0 indicated that the cat was interactive and interested when approached and did not mind clinical examination; a score of 1 referred to a cat that was scared or nervous, hid at the back of the kennel when approached, or tolerated clinical examination but remained rigid and hid against the walls; and score 2 represented aggressive behavior, such as biting or scratching, that precluded some or all of the clinical examination [37].

Heart rate (HR), respiratory rate (RR), and rectal temperature were recorded at baseline and after 30 min, 1, 2, 4, 8, 12, and 24 h. HR was measured by stethoscope and RR by observation of thoracic excursions, both of which were measured over a 30 s interval. Rectal temperature was measured after all other behavioral and physiological evaluations were performed to avoid the potential influence of thermometer insertion on these variables. A 2 mL venous blood sample was collected from each cat through an intravenous catheter placed in the cephalic vein 1, 2, 4, and 12 h after CBD administration for subsequent analysis of CBD plasma concentrations. Blood chemical analyses were conducted 24 h and 2 weeks post administration to assess changes in blood chemistry relative to pre-dosing values.

Mechanical sensitivity was assessed using von Frey filaments (Aesthesio^®^; Danmic Global LLC, San Jose, CA, USA). The smallest diameter filament (0.08 g) was applied perpendicularly to the skin at the lumbosacral joint area until the filament bent or a positive response was observed. Positive responses included the head turning toward the application site, back skin contraction, or movement away from the device. If no response was detected, the next larger filament was applied. This process continued until a positive response was observed or the largest filament (300 g) was used. The filament that first elicited a positive response at least two times in three repeated measurements, with 30 s intervals between applications, was recorded.

Mechanical nociceptive thresholds were measured using a hand-held algometer (Dolorimeter; Fabrication Enterprises Inc., White Plains, NY, USA) equipped with 0.31 cm^2^ flat circular probe and a 5-pound (2.27 kg) capacity gauge. The algometer was applied perpendicularly to three anatomical sites: the lumbosacral joint area and the medial aspects of the left and right stifle joints, specifically between the patella and the tibial tuberosity. These measurement sites were selected based on a previous study in cats [38]. The order of application (lumbosacral area, left stifle, or right stifle) was randomized for each cat. Pressure was applied gradually until an evoked response was observed. Each site was tested three times, with 30 s intervals between applications. The pressure that first elicited a response was recorded and averaged. Evoked responses included limb or back withdrawal, standing up to walk away, vocalization, hissing, or attempts to bite or scratch. All threshold measurements were performed after the completion of behavioral observations, physiological assessments, and mechanical sensitivity tests. A single investigator conducted all measurements using both von Frey filaments and the algometer to ensure consistency across all cats. Both devices were factory-calibrated and did not require recalibration prior to use. However, to verify measurement accuracy, the algometer probe was pressed against a calibrated digital weighing scale, and incremental forces of 0.5, 1.0, 1.5, and 2.0 kg were applied. The scale readings consistently matched the applied forces, confirming consistent device output. Although no standardized external calibration protocol was available for this veterinary-specific device, consistency across test readings and the use of a single trained evaluator were employed to ensure measurement reliability.

Statistical analyses were performed using NCSS statistical software (version 21.0.2) and R (version 4.3.2). Data were summarized using descriptive statistics, with continuous variables presented as the mean ± SD for normally distributed data or as the median with interquartile range (IQR) for non-normally distributed data. The Shapiro–Wilk test was used to assess data normality. Repeated-measures analysis of variance (ANOVA), followed by Tukey–Kramer multiple comparison tests, was employed to compare differences between the baseline and other timepoints for parametric data. For non-parametric data, the Friedman test was conducted to assess differences over time, followed by Dunn’s post hoc test with Benjamini–Hochberg correction to adjust for multiple comparisons and control the false discovery rate (FDR). Statistical significance was set at α = 0.05.

### 2.3. Quantification of CBD Concentrations in Plasma via In-Solution Digestion and Nanoscale Liquid Chromatography–Tandem Mass Spectrometry (nanoLC-MS/MS)

Blood samples collected in heparin tubes were centrifuged to separate the plasma, which was then aliquoted and stored at −80 °C until analysis. Plasma total protein levels were determined using Lowry’s assay, with bovine plasma albumin as the reference standard [39]. Protein samples were prepared by reducing disulfide bonds with 5 mM dithiothreitol at 60 °C for 1 h, followed by alkylation with 15 mM iodoacetamide at 25 °C for 45 min. Proteins were then digested with trypsin for 3 h at room temperature, and the resulting peptides were dissolved in 0.1% formic acid for LC-MS/MS analysis.

Peptide identification was conducted using the Ultimate 3000 Nano/Capillary LC System coupled with a ZenoTOF 7600 mass spectrometer. Digested peptides were con-centrated on a C18 µ-Precolumn and separated using a C18 RSLC column at 35 °C with a gradient of 5–55% solvent B (0.1% formic acid in 80% acetonitrile) over 30 min at 0.30 µL/min. The ZenoTOF 7600 operated in positive polarity with optimized source parameters and selected the top 50 precursor ions (≥150 cps) for MS/MS analysis in a 3.0 s cycle, applying dynamic exclusion for 12 s. MS2 spectra were acquired over 100–1800 *m*/*z* with a 50 ms accumulation time. Quality control included triplicate sample analysis for reproducibility and bovine plasma albumin digestion as a performance check [40].

### 2.4. Bioinformatic Analysis of CBD Concentration in Plasma

For protein identification, raw mass spectral data were analyzed using MaxQuant software (version 2.2.0.0) to identify peptides and proteins. MS/MS data were searched against the reference proteome database for the target *Cannabis sativa* (Hemp) (Mari-juana), obtained from UniProt and supplemented with a contaminant database [41]. Protein identification was considered significant with a *p*-value < 0.05 and a 1% FDR applied to both peptides and proteins. Peptides were required to have a minimum length of 7 amino acids and at least one unique peptide, adhering to established protocols [42,43]. Label-free quantification was performed using the MaxLFQ algorithm in MaxQuant, with a minimum ratio count of 2. CBD-associated proteins in cat plasma were searched by targeting “tetrahydrocannabinolic acid (THCA) synthase and cannabidiolic acid (CBDA) synthase” from the protein list. A comparison of THCA/CBDA synthase proteins and reviewed entries “Cannabis” (116 proteins; Table S1) in the UniProt Swiss-Prot database (24 December 2024) was visualized using a Jvenn diagram [44]. Statistical analysis was conducted using ANOVA with Fisher’s exact test to identify significant proteins (*p* < 0.05) across datasets.

## 3. Results

### 3.1. Behavioral Observations and Physiological Data

**Figure 1:**
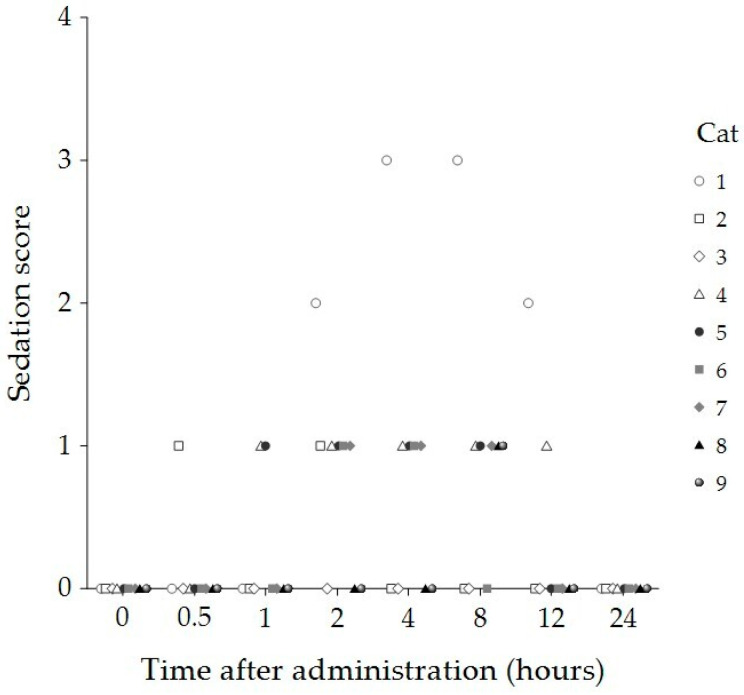
Sedation scores of individual cats (n = 9) at baseline (0 h) and 30 min, 1, 2, 4, 8, 12, and 24 h after administration of 8 mg/kg CBD oil. Each symbol represents a single cat.

Sedation, compliance, and temperament scores for the nine cats at baseline and 30 min, 1, 2, 4, 8, 12, and 24 h following administration of 8 mg/kg of CBD oil are summarized in Table 1. Statistically significant increases in sedation scores were observed 2, 4, and 8 h post administration compared to baseline (*p* < 0.001). Specifically, after 2 h, three cats remained fully awake and able to stand and walk (sedation score = 0), five cats exhibited staggering when attempting to walk (score = 1), and one cat showed sternal recumbency but was able to lift its head (score = 2). After 4 h, four cats had a sedation score of 0, four scored 1, and one cat developed lateral recumbency but responded to stimulation (score = 3). By 8 h, three cats had a sedation score of 0, five scored 1, and one scored 3 (Figure 1).

**Figure 2:**
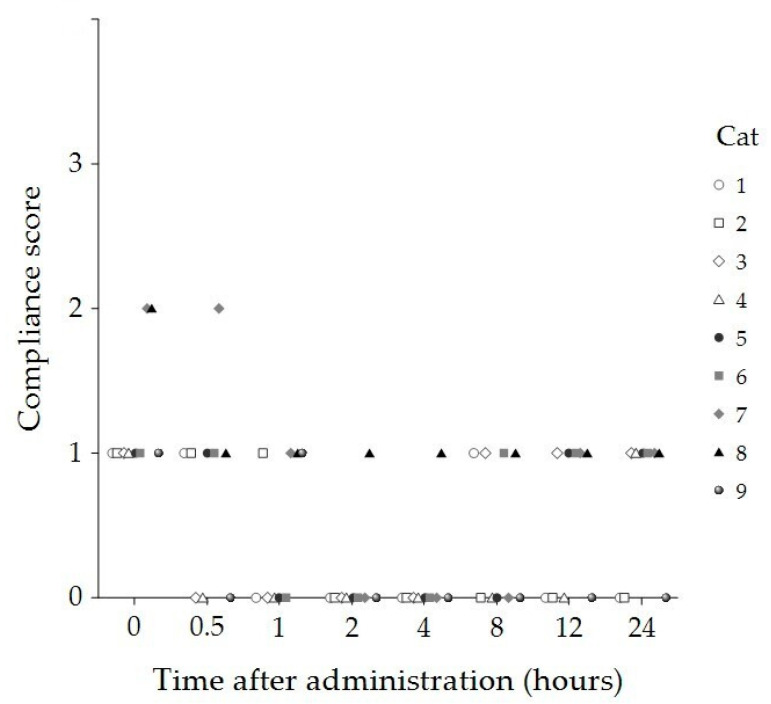
Compliance scores of individual cats (n = 9) at baseline (0 h) and 30 min, 1, 2, 4, 8, 12, and 24 h following administration of 8 mg/kg CBD oil. Each symbol represents a single cat.

**Figure 3:**
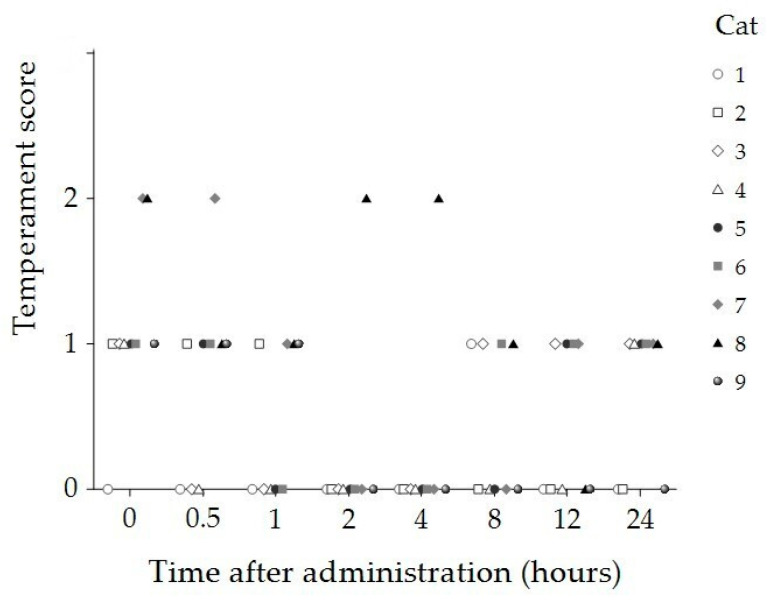
Temperament scores of individual cats (n = 9) at baseline (0 h) and 30 min, 1, 2, 4, 8, 12, and 24 h following administration of 8 mg/kg CBD oil. Each symbol represents a single cat.

Compliance and temperament scores significantly decreased 2 and 4 h post administration compared to baseline (*p* < 0.001 and *p* = 0.012, respectively) (Table 1). At both timepoints, eight cats displayed no resistance to handling (compliance score = 0) and remained calm throughout the physiological assessments (temperament score = 0). In contrast, one cat demonstrated minimal resistance to handling (compliance score = 1) and exhibited aggressive behaviors during the assessments (temperament score = 2) at both timepoints (Figure 2 and Figure 3).

Physiological parameters, including HR, RR, and body temperature, did not show significant changes over time following CBD administration, as demonstrated in Table 2. Hypersalivation was observed in four cats immediately after oral CBD administration. Drowsiness, characterized by drooping eyelids and signs of lethargy, was noted in eight cats at least once between 30 min and 4 h post administration. Two cats experienced a single episode of vomiting after receiving CBD. One Persian cat exhibited redness of the ear pinna and increased water consumption 4 and 8 h post administration. AST was the only blood chemistry parameter that showed a significant increase 24 h post administration, which returned to the baseline level when re-evaluated 2 weeks post dosing (Table 3).

### 3.2. Mechanical Sensitivity and Nociceptive Thresholds

One cat was excluded from mechanical sensitivity testing with von Frey filaments due to a complete absence of responses to all filament sizes at baseline and throughout all post-treatment timepoints. Two cats were excluded from the mechanical nociceptive threshold testing because they exhibited strong aversive reactions immediately upon contact with the algometer, preventing further assessment.

**Figure 4:**
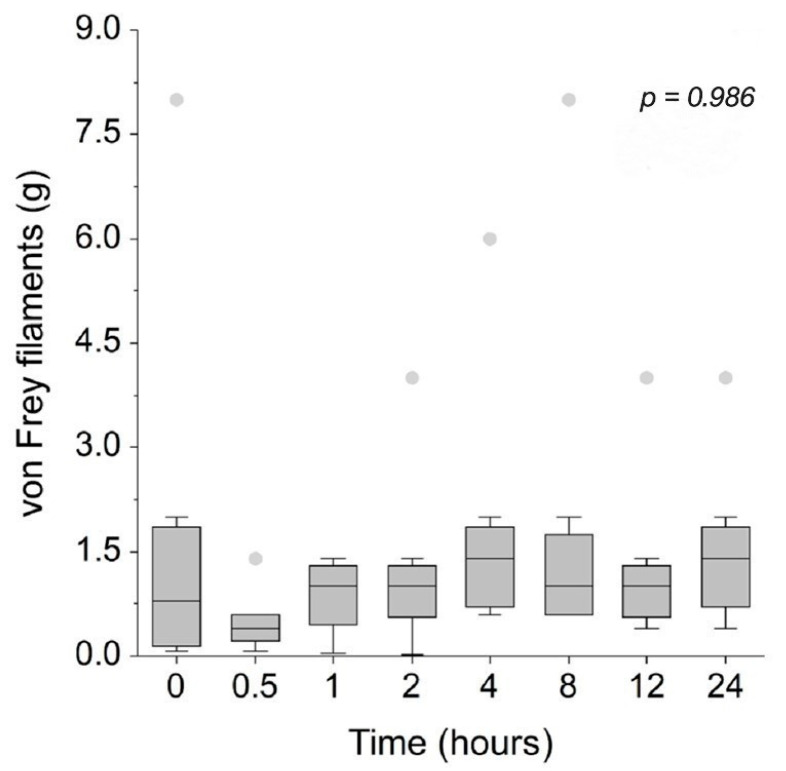
Mechanical sensitivity measured using von Frey filaments at the lumbosacral joint area of eight cats at baseline (0 h) and 30 min, 1, 2, 4, 8, 12, and 24 h after administration of 8 mg/kg CBD. The horizontal line within each box represents the median. Box limits correspond to the 25th and 75th percentiles, and whiskers extend to the minimum and maximum values within 1.5 times the interquartile range (IQR). Outliers beyond this range are displayed as individual markers.

**Figure 5:**
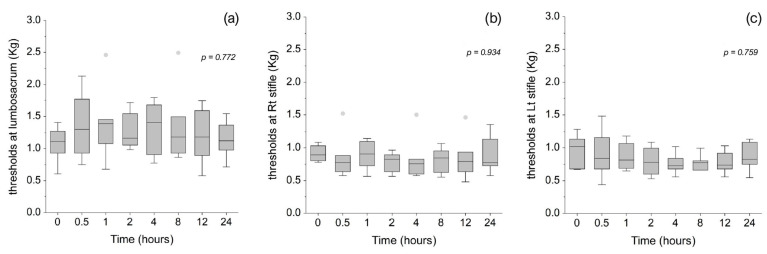
Mechanical nociceptive thresholds measured using an algometer at the lumbosacral joint area (**a**), the medial aspect of the right stifle joint (**b**), and the medial aspect of the left stifle joint (**c**) in seven cats at baseline (0 h) and 30 min, 1, 2, 4, 8, 12, and 24 h after administration of 8 mg/kg CBD. The horizontal line within each box represents the median. Box limits indicate the 25th and 75th percentiles, and whiskers extend to the minimum and maximum values within 1.5 times the interquartile range (IQR). Data points beyond this range are considered outliers and are displayed as individual markers.

Mechanical sensitivity at the lumbosacral area, as measured by von Frey filaments and illustrated in Figure 4, remained unchanged from baseline across all timepoints following CBD administration. Nociceptive thresholds measured using an algometer at three anatomical sites—the lumbosacral area (Figure 5a), medial aspect of the right stifle joint (Figure 5b), and medial aspect of the left stifle joint (Figure 5c)—also showed no significant changes compared to baseline throughout the observation period. However, it is important to note that the exclusion of some cats directly reduces the sample size and may have limited the statistical power to detect subtle changes in nociceptive thresholds following CBD administration.

### 3.3. Plasma Concentrations

**Figure 6:**
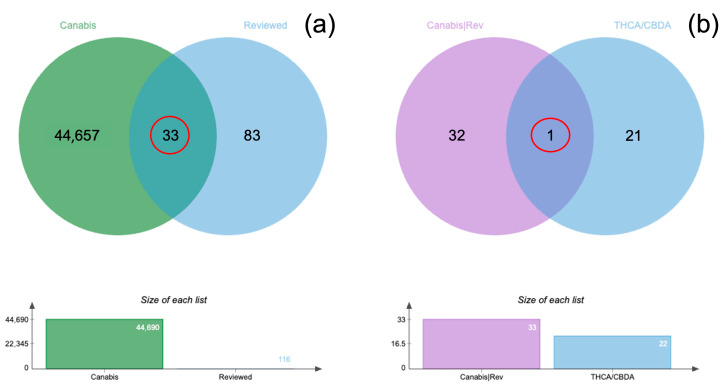
The Venn diagram compares all identified proteins labeled as “Cannabis” (44,657 proteins) with “Reviewed” entries (116 proteins). The intersection shows 33 proteins that are common between these two datasets, indicating proteins associated with cannabis and also reviewed entries (**a**). The Venn diagram displays the distribution of the 33 “Cannabis/Reviewed” proteins compared to THCA and CBDA synthase proteins (22 proteins in total). A single protein is common between these datasets (**b**). Below each Venn diagram, bar graphs illustrate the size of each dataset: A: “Cannabis” (44,690) and “Reviewed” (116); B: “Cannabis/Reviewed” (33) and “THCA/CBDA synthase” (22).

The 44,698 proteins identified from all samples were analyzed using in-solution di-gestion coupled with LC-MS/MS (Table S2). A total of 33 proteins were found to be common between all identified proteins and the reviewed entries labeled as “Cannabis,” as illustrated by the Venn diagram (Figure 6a; Table S3). Among all identified proteins, 14 THCA synthase proteins and 8 CBDA synthase proteins were detected (Table S4). The comparison of these THCA/CBDA synthase proteins with the 33 common “Cannabis/Reviewed” proteins revealed a single overlapping protein, identified as cannabidiolic acid synthase-like 2 (CBDAS3) (Figure 6b). Furthermore, CBDAS3 and four additional THCA/CBDA synthase proteins with gene names—including tetrahydrocannabinolic acid synthase (THCA), THCA4, THCA5, and THCAS—were analyzed for their concentrations in triplicate using MaxQuant software (Table S5).

**Figure 7:**
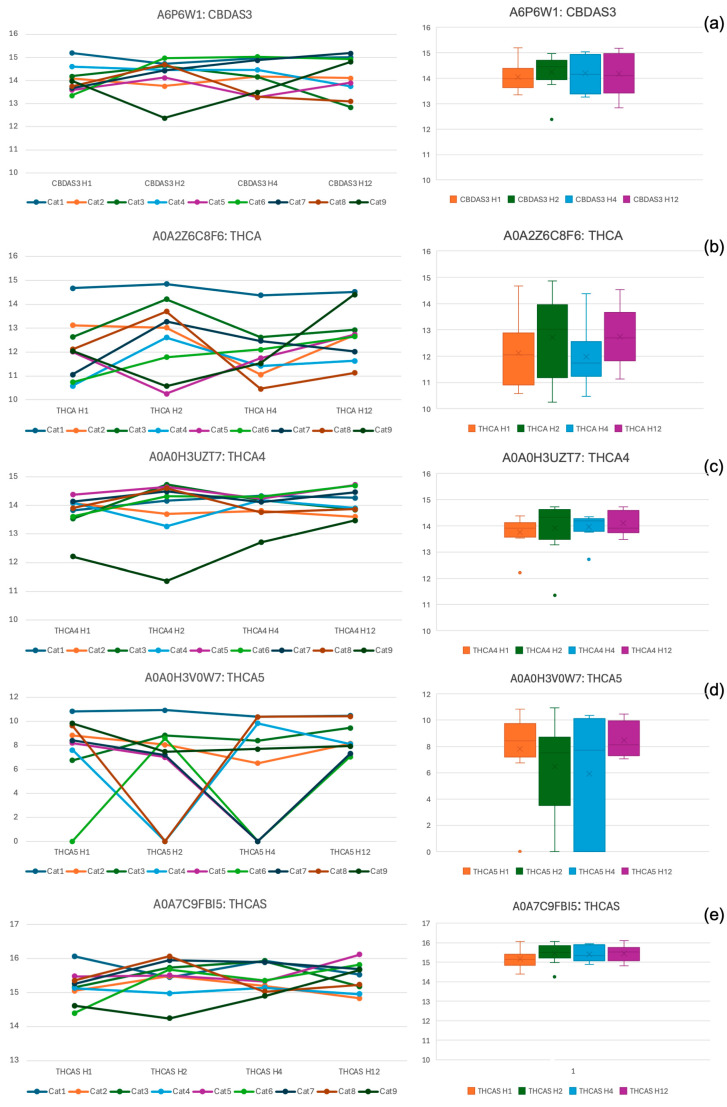
Line graphs illustrating the concentrations of five cannabis-related proteins (CBDAS3 (**a**), THCA (**b**), THCA4 (**c**), THCA5 (**d**), and THCAS (**e**)) in each cat 1 h (H1), 2 h (H2), 4 h (H4), and 12 h (H12) after administering 8 mg/kg of CBD oil. Box plots presenting the median concentrations of the same five cannabis-related proteins (CBDAS3 (**a**), THCA (**b**), THCA4 (**c**), THCA5 (**d**), and THCAS (**e**)), along with their interquartile ranges (IQR), across the four timepoints (1, 2, 4, and 12 h). The median concentrations remained consistent over time, with no statistically significant differences observed at any post-administration timepoint (*p* > 0.05).

The median concentrations of five cannabis-related proteins, as measured by mass spectrometry, were analyzed in nine cats 1, 2, 4, and 12 h following the administration of 8 mg/kg of CBD. These results are depicted in Figure 7a–e, with individual protein concentrations displayed using line graphs and the median values with IQR shown in box plots.

No statistically significant differences in the concentrations of the five cannabis-related proteins were observed at any post-administration timepoints (1, 2, 4, and 12 h) (*p* > 0.05). However, one cat (Cat1) consistently exhibited notably high concentrations of THCA and THCA5 across the four post-dosing timepoints.

## 4. Discussion

The findings of this study indicate that oral administration of 8 mg/kg CBD oil influences feline behavior, particularly with respect to sedation, compliance, and temperament. CBD appears to induce sedation in cats, as indicated by significantly increased sedation scores between 2 and 8 h post administration, suggesting a relatively long-lasting sedative effect. The sedation was generally mild, with a median score of 1, typically characterized by staggered walking. However, this effect may have been influenced by stress due to temporary separation from caregivers and exposure to an unfamiliar environment, which may have elevated the baseline anxiety levels of the study cats. Therefore, under less stressful conditions, the degree, onset of action, and duration of sedation might differ. Further studies examining the impact of environmental stress on CBD-induced sedation would enhance understanding of its clinical applicability in cats.

In addition, individual variability was observed, as a 1-year-old domestic shorthair (Cat1) exhibited deeper sedation (scores of 2 to 3) that persisted for up to 12 h post dosing. These findings underscore the importance of accounting for individual differences when determining appropriate CBD dosages for clinical use in cats. In parallel, LC-MS/MS analysis detected specific cannabinoids, including cannabidiolic acid synthase-like 2 (CBDAS3) and tetrahydrocannabinolic acid synthases (THCA, THCA4, THCA5, and THCAS), in plasma samples. Notably, Cat #1 exhibited relatively higher plasma concentrations of THCA and THCA5 during the post-administration period compared to the other cats, coinciding with higher sedation scores and a prolonged duration of effect. This temporal association raises a speculative hypothesis; however, no causal inference can be made regarding the potential contribution of THCA and THCA5 to sedation.

Of note, the detection of cannabidiolic acid synthase-like 2 and THCA synthases—enzymes typically involved in cannabinoid biosynthesis in plants [46]—raises questions regarding their presence in feline plasma following CBD administration. As these enzymes are not endogenously expressed in mammals, their detection likely reflects passive absorption of plant-derived proteins rather than any functional involvement in metabolism. While the biological significance of their presence remains unclear, this exploratory finding underscores the need for continued investigation. Given the complexity of cannabinoid metabolism and the proteomic detection of these enzymes, further research is warranted to determine whether they play any functional role in pharmacokinetics, pharmacodynamics, or cannabinoid efficacy in animals.

The sedative effect of CBD observed in cats in this study is consistent with findings in mice, where CBD exerted sedative and hypnotic effects at doses of 10 mg/kg and 20 mg/kg. These effects were evidenced by increased sleep duration, reduced sleep latency in both normal and insomnia-induced mice, and a significant reduction in locomotor activity at the 20 mg/kg dose compared to controls [47]. In that study, activation of 5-HT_1A_ receptors was proposed as one of the potential mechanisms, as the sedative–hypnotic effects of CBD were inhibited in mice pre-treated with a 5-HT_1A_ receptor antagonist [47].

While significant sedation was detected, there were no discernible changes in physiological parameters such as HR, RR, and body temperature throughout the study period. This finding is consistent with previous reports in humans, rats, mice, and horses, which have shown no adverse effects of various CBD concentrations on these physiological parameters [48,49]. These results imply that CBD at the administered dose may produce a sedative effect without causing significant physiological disturbances in healthy cats. This is particularly relevant in veterinary practice, where minimizing adverse effects on cardiovascular and respiratory function is essential when selecting sedation options for animals. Nevertheless, it is important to note that more accurate and reliable methods for assessing cardiovascular and respiratory function, such as blood pressure measurement, electrocardiography, and arterial blood gas analysis, were not utilized in this study. As such, further research remains warranted to thoroughly evaluate the potential side effects of CBD on the cardiovascular and respiratory systems.

A decrease in compliance and temperament scores 2 and 4 h post administration indicated a reduction in the cats’ resistance to handling and assessments. These results may reflect sedative-induced lethargy, as the sedation level increased during the corresponding time periods. Additionally, CBD administration may enhance the emotional well-being of cats by reducing stress and inducing a state of calm, as the anxiolytic properties of CBD have been demonstrated in previous studies. For example, dogs [19] and cats [20] have shown reduced anxiety during separation from caregivers after a 2-week period of daily CBD administration. In mice, CBD at a dose of 3 mg/kg was shown to mitigate stress-induced hyperthermia [50]. These findings support the potential therapeutic application of CBD in managing stress and improving compliance during handling in veterinary patients. In practical terms, such effects may be particularly beneficial during routine procedures that often provoke fear or resistance in animals, such as blood sampling, intramuscular injection, or intravenous catheter placement. The mild sedation observed may also reduce the need for additional restraint or chemical sedation, thereby improving both patient welfare and procedural efficiency. However, species-specific responses, behavioral conditions, and variations in dose regimens must be considered. As an example, CBD treatment in shelter dogs with behavioral disorders did not lead to behavioral improvement and was still associated with a significant increase in hair cortisol levels following exposure to common stressors [51]. These contrasting findings highlight the complex and context-dependent effects of CBD across different species and physiological states. According to plasma cannabinoid analysis, although the levels of detected THCA synthases and CBDAS3 did not significantly differ across timepoints, most exhibited a slight increasing trend after 2 h compared to 1 h post administration. While this trend was temporally associated with notable changes in compliance, temperament, and sedation scores, further investigation with a larger sample size is needed to clarify any potential association between these cannabinoids and behavioral effects in cats.

Interestingly, the observed time course of significant changes in the level of sedation, compliance, and temperament aligns with previously published pharmacokinetic data, which indicate that peak CBD plasma concentrations occur between 2 and 2.6 h following the administration of various doses, such as 1.37, 2, 2.5, 5, 10, 20, and 40 mg/kg of CBD in cats [23,25,26,52]. This information can be useful in guiding both owners and veterinary staff to wait for 2 h after CBD administration to ensure that the drug reaches its maximum effect, thereby increasing the likelihood of successful clinical interactions with stressed cats.

A single dose of 8 mg/kg CBD did not result in significant changes in mechanical sensitivity and nociceptive thresholds in cats, as both von Frey filament and algometer testing produced results consistent with baseline measurements across all post-administration timepoints. These findings diverge from prior studies that indicated antinociceptive and analgesic properties of CBD in animal models. In canine osteoarthritis studies, administration of 2 mg/kg CBD twice daily in various breeds and 50 mg/day in large domestic dogs reduced pain scores, increased comfort and activity levels, and improved overall quality of life [17,18]. In a Parkinson’s disease model, parkinsonism-induced mice exhibited reduced thermal thresholds in hot plate and tail flick tests and lower mechanical nociceptive thresholds in von Frey filament tests, while intraperitoneal CBD at doses of 10 and 100 mg/kg increased these thresholds, alleviating hyperalgesia and allodynia [53]. In a neuropathic pain rat model, 3 mg/kg of intraperitoneal CBD effectively reversed mechanical and thermal allodynia, hyperalgesia, and associated anxiety-like behaviors [54]. Moreover, in a rat arthritis model, transdermal CBD at doses of 6.2 and 62 mg/day significantly reduced joint swelling and spontaneous pain scores, while restoring paw withdrawal latency to near-baseline levels in response to noxious heat stimulation [55].

The absence of antinociceptive effects observed in the present study may be attributed to the context-dependent nature of CBD’s efficacy in pain modulation. Factors such as the type of pain (e.g., nociceptive, acute, chronic, inflammatory, neuropathic pain), each with distinct underlying mechanisms, along with dosage regimens and interspecies differences, may contribute to this variability. It is possible that the use of healthy cats without an induced pain condition in this study may not have activated the relevant pain pathways that CBD is more likely to modulate. This observation is compatible with the known pathophysiological roles of cannabinoid receptors in neurological and inflammatory diseases [7], as well as CBD’s interactions with non-cannabinoid receptors, such as 5-HT_1A_ and TRPV1, which are particularly implicated in inflammatory states [8,9,10,11,12]. Consequently, CBD’s antinociceptive effects may be more pronounced in pathological pain conditions, contrasting with its limited impact under normal physiological states.

Another important consideration is the duration of CBD exposure and the potential for delayed or cumulative effects, as observed in studies on canine osteoarthritis, where analgesic outcomes were reported after 2 and 4 weeks of daily treatment [17,18]. This contrasts with the present study, which assessed effects following a single dose. Longer-term use or higher dosing (e.g., greater than 8 mg/kg) may yield different outcomes. Therefore, exploring extended treatment protocols or alternative regimens could provide deeper insights into CBD’s potential for pain modulation in cats. In addition, interspecies differences in cannabinoid receptor distribution may contribute to the limited antinociceptive response observed in cats. For instance, dogs have been shown to possess a significantly higher number of CB1 receptors in hindbrain structures compared to humans [4], suggesting that receptor localization and density could influence the pharmacodynamic effects of cannabinoids across species. While comparable data for cats remain limited, such anatomical differences may partially explain the variability in analgesic efficacy observed between feline and non-feline models.

Furthermore, certain constraints should be considered when interpreting the mechanical testing results. One and two cats were excluded from sensitivity and threshold testing, respectively, due to unexpected responses. These exclusions may have reduced the statistical power and limited the ability to detect potential treatment effects on mechanical sensitivity and nociceptive thresholds. One cat exhibited a complete absence of response to von Frey filament testing at all timepoints, possibly due to fear-induced immobility or hyposensitivity stemming from an undetected condition during the initial health screening. The other two displayed strong aversive reactions to the algometer upon contact. These reactions underscore differences in sensitivity to mechanical stimuli among cats. This variation may have been influenced by the diverse backgrounds of the cats, as they were sourced from different owners and had no prior exposure to or familiarity with experimental equipment or environments. Such unfamiliarity may have contributed to fear, hyperexcitement, or hyperactivity. To minimize individual disparities in mechanical sensitivity and nociceptive threshold testing, future studies could benefit from using cats raised in a standardized experimental setting, with more uniform characteristics such as age and breed. These findings also highlight the limited feasibility of using certain nociceptive assessment tools under conditions where animals are unfamiliar with the testing setup. Further refinement and validation of these methods are necessary to improve their applicability in feline studies.

Previously reported adverse effects of CBD oil in cats include drowsiness, lethargy, excessive salivation, and elevated liver enzymes [23,24,25,26,27]. In the present study, hypersalivation, emesis, drowsiness, and erythema of the ear pinnae were observed, emphasizing the need for careful consideration when prescribing CBD oil for feline patients. A transient elevation in AST above the reference range was detected 24 h post administration but returned to baseline by two weeks after treatment. The observed increase may be associated with the liver’s metabolic processing of CBD via cytochrome P450 enzymes, indicating elevated hepatic activity rather than actual liver damage [56]. However, caution is advised when using CBD oil in cats with pre-existing hepatic conditions. Hypersalivation occurred immediately after administration in 44% of cats, possibly due to the taste of the capsule or leakage from a broken capsule. Vomiting occurred as a single event in two cats; no diarrhea or soft stool was reported. The episodes of vomiting may be attributed to individual sensitivity to the taste or odor of the CBD oil, irritation from the capsule contents if rupture occurred orally, or gastrointestinal intolerance to the carrier oil. While the CBD–THC ratio of ≥20:1 minimizes psychoactive effects, trace THC content may also contribute to nausea or emesis in susceptible individuals [56]. Such reactions underscore the importance of formulation palatability and delivery method in feline patients. Drowsiness, observed in 89% of cats, was the most prominent effect and may be related to the sedative–hypnotic properties of CBD. Erythema of the ear pinnae and increased water intake were noted in one cat, potentially indicating hypersensitivity or an allergic reaction to the CBD oil. However, it remains unclear whether the reaction was triggered by the CBD itself or by the carrier oil used as a vehicle. Although these adverse effects were generally mild and transient, they raise potential concerns about the tolerability of the 8 mg/kg dose used. Careful dose titration and monitoring are therefore necessary to minimize the risk of undesirable side effects.

These findings underscore the need for careful consideration when prescribing CBD oil for feline patients, particularly in light of potential adverse effects such as emesis, hypersalivation, and elevated AST levels. Given the increasing accessibility of CBD products for pets and the potential for misuse or misinformed application, it is essential to communicate evidence-based information to the broader public. Social media may serve as an effective tool in this regard; for example, a recent study demonstrated how platforms such as Instagram can help convey complex scientific topics—such as safety concerns and the behavioral and physiological effects of CBD—to a wider audience [57].

Limitations of this study should be acknowledged. First, the scaling systems used to assess sedation, temperament, and compliance in cats were not formally validated prior to use, and interpretations were not guided by veterinary behavior specialists. However, these systems have been employed in previous studies investigating the effects of opioids and other central nervous system depressants in this species [32,33,34,35], and consistency was maintained by using a single trained evaluator throughout. Future studies should incorporate expert input and formal validation to enhance the reliability of behavioral assessments. Second, the temperament score combined signs of fear and aggression into a single ordinal scale, which may have masked important distinctions in the cats’ behavioral responses to CBD. Future studies should consider separating these emotional responses to better elucidate the specific effects of CBD on feline behavior. Third, the cats were not acclimated to the study environment or equipment before testing began. This approach aimed to establish a consistent baseline by ensuring that all cats—despite originating from diverse household backgrounds—were housed overnight in the same novel environment and separated from their owners prior to the study. This was intended to standardize initial conditions while still reflecting the variability typically seen in real-world clinical populations. However, the lack of acclimatization may have contributed to unexpected responses during mechanical testing, as previously discussed, and some of the observed behavioral responses may have reflected environmental stress rather than pharmacological effects alone. Fourth, the preliminary dose-finding data were derived from a small number of animals. While useful for estimating the CBD dose, the limited sample size reduces the strength and generalizability of these findings. This issue extended into the main study, where financial constraints also led to a modest sample size and the absence of a control group. These limitations reduce the generalizability of the findings and warrant cautious interpretation. In the absence of a placebo group, the potential influence of other factors—such as environmental stress and circadian or repeated handling influences—on behavior and sedation cannot be ruled out. Future studies employing randomized, placebo-controlled designs are necessary to validate the behavioral and physiological effects observed in this investigation.

Another limitation of this study lies in the absence of global multiple comparison correction across all endpoints. This raises the potential risk of inflated Type I error due to the large number of comparisons. However, given the exploratory nature of this study and the fact that these outcomes represent distinct biological domains evaluated independently, a within-family correction (e.g., Benjamini–Hochberg) was applied within each outcome domain to balance Type I error control with statistical sensitivity. Applying a more conservative global adjustment (e.g., Bonferroni across all comparisons) would likely have increased the risk of Type II error, reducing the ability to detect potentially meaningful effects in this exploratory, multi-domain investigation [58,59]. Nonetheless, this methodological choice should be acknowledged when interpreting the significance of individual findings. Moreover, although the CBD product used in this study contained only trace amounts of THC (CBD–THC ≥ 20:1), the possibility that these minimal levels contributed to the observed effects or adverse outcomes cannot be entirely excluded. This consideration is particularly relevant given the plasma detection of THCA and THCAS, which may suggest some degree of biological activity even at low concentrations. Additionally, the limited number of plasma sampling timepoints may have hindered the detection of more detailed pharmacokinetic patterns. Future studies should incorporate more frequent sampling and larger sample sizes, both to validate the current findings and to facilitate the identification of cannabis-related proteins potentially involved in behavioral modulation, which could offer valuable insights for therapeutic development.

## 5. Conclusions

In conclusion, this study demonstrates that oral administration of CBD at 8 mg/kg induces mild sedation in healthy cats, reducing resistance during handling and assessments without significantly altering HR, RR, or body temperature, but it does not appear to affect mechanical sensitivity or nociceptive thresholds under non-painful conditions. These results support CBD’s potential application in veterinary settings, particularly for non-painful procedures where mild sedation and improved compliance are beneficial. However, as these observations remain exploratory—given the small sample size and absence of a control group—randomized, placebo-controlled trials with larger cohorts are warranted to validate these findings.

## Figures and Tables

**Table 1:** Sedation, temperament, and compliance scores of nine cats at baseline (prior to administration) and 30 min, 1, 2, 4, 8, 12 and 24 h after receiving 8 mg/kg of CBD oil.

Time (Hours)	Sedation Score Median (IQR)	Compliance Score Median (IQR)	Temperament Score Median (IQR)
Baseline	0 (0, 0)	1 (1, 1.5)	1 (1, 1.5)
1/2	0 (0, 0)	1 (0, 1)	1 (0, 1)
1	0 (0, 0.5)	0 (0, 1)	0 (0, 1)
2	1 (0, 1) *	0 (0, 0) *	0 (0, 0) *
4	1 (0, 1) *	0 (0, 0) *	0 (0, 0) *
8	1 (0, 1) *	0 (0, 1)	0 (0, 1)
12	0 (0, 0.5)	1 (0, 1)	0 (0, 1)
24	0 (0, 0)	1 (0, 1)	1 (0, 1)
*p*-value	<0.001	<0.001	0.012

* Indicates a significant difference compared to the baseline value. IQR = interquartile range.

**Table 2:** Heart rate (HR), respiratory rate (RR), and body temperature in nine cats at baseline (prior to administration) and 30 min, 1, 2, 4, 8, 12, and 24 h following administration of 8 mg/kg CBD oil.

Time (Hours)	HR (Beats/Minute) Median (IQR)	RR (Breaths/Minute) Median (IQR)	Temperature (°C) Median (IQR)
Baseline	168 (160, 190)	32 (22, 61)	38.1 (37.8, 39)
1/2	180 (166, 200)	26 (24, 31)	38.0 (37.8, 38.4)
1	180 (168, 190)	28 (22, 64)	37.9 (37.7, 38.6)
2	180 (160, 200)	28 (22, 32)	37.9 (37.7, 39.1)
4	180 (160, 190)	24 (20, 26)	37.9 (37.7, 38.2)
8	180 (164, 194)	26 (20, 36)	37.7 (37.2, 38.3)
12	184 (176, 200)	24 (22, 27)	37.8 (37.8, 38.2)
24	180 (160, 180)	24 (20, 24)	37.8 (37.8, 38.1)
*p*-value	0.792	0.182	0.232

IQR = interquartile range, and °C = degrees Celsius.

**Table 3:** Mean and standard deviation (mean ± SD) of blood chemistry profiles at baseline (prior to administration) and 1 day (24 h) and 2 weeks following administration of 8 mg/kg CBD oil in cats.

Blood Chemistry	Baseline (Pre-CBD)	1 Day Post-CBD	2 Weeks Post-CBD	*p*-Value	Reference Interval **
BUN (mg/dL)	27 ± 5.37	24 ± 5.64	26 ± 4.52	0.052	19–34
Creatinine (mg/dL)	1.45 ± 0.27	1.37 ± 0.34	1.35 ± 0.30	0.376	0.9–2.2
ALT (U/L)	40.27 ± 16.40	49.22 ± 13.57	48.33 ± 13.80	0.134	25–97
ALP (U/L)	35.89 ± 17.74	42.33 ± 21.18	38.78 ± 17.03	0.179	0–45
AST (U/L)	28.22 ± 7.36	48.89 ± 27.22	26.22 ± 6.94	0.018 *	7–38
Total protein (g/dL)	7.30 ± 0.52	7.00 ± 0.44	6.92 ± 0.45	0.201	6–7.9
Albumin (g/dL)	3.52 ± 0.21	3.41 ± 0.22	3.40 ± 0.28	0.651	2.8–3.9

BUN = blood urea nitrogen, ALT = alanine aminotransferase, ALP = alkaline phosphatase, and AST = aspartate aminotransferase. * Statistically significant at *p* < 0.05. ** Reference limits were determined based on veterinary laboratory guidelines [45].

## Data Availability

The data presented in this study are available in the article. Additional raw data are available from the corresponding author upon request. The MS/MS raw data and analysis are available in the ProteomeXchange Consortium (accessed on 30 May 2025) via the jPOST partner repository: JPST003838 and PXD064409.

## Supplementary Materials

The following supporting information can be downloaded at: https://www.mdpi.com/article/10.3390/ani15131987/s1, Table S1: The list of reviewed entries “Cannabis” 116 proteins in the UniProt Swiss-Prot database (24 December 2024); Table S2: The list of 44,698 proteins identified from all plasma samples were analyzed using in-solution digestion coupled with LC-MS/MS; Table S3: The list of common between all identified proteins and the reviewed entries labeled as “Cannabis,” as illustrated by the Venn diagram; Table S4: The list of 14 THCA synthase proteins and 8 CBDA synthase proteins were selected from all identified proteins; Table S5: The list of five THCA and CBDA synthase proteins identified in cat serum with gene names and their concentrations in triplicate using MaxQuant software.

## Abbreviations

The following abbreviations are used in this manuscript:

CBD	Cannabidiol
ECS	Endocannabinoid system
CB1	Cannabinoid receptors 1
CB2	Cannabinoid receptors 2
5-HT_1A_	Serotonin 5-HT_1A_ receptor
TRPV1	Transient receptor potential vanilloid 1
THC	Tetrahydrocannabinol
nanoLC-MS/MS	Nanoscale liquid chromatography–tandem mass spectrometry
BUN	Blood urea nitrogen
ALT	Alanine aminotransferase
AST	Aspartate aminotransferase
DSH	Domestic shorthair
SD	Standard deviation
HR	Heart rate
RR	Respiratory rate
IQR	Interquartile range
ANOVA	Analysis of variance
FDR	False discovery rate
MS2	Tandem mass spectrometry
THCA	Tetrahydrocannabinolic acid
CBDA	Cannabidiolic acid

## Appendix A

**Table A1:** Numerical rating scale for sedation, adapted from established methods used in feline sedation assessment [32,33,34,35].

Criteria	Score
Completely awake, able to stand and walk, normal posture	0
Stands but staggers when attempting to walk	1
Sternal recumbency, able to lift head up, unable to stand, may makes weak attempts to rise	2
Lateral recumbency, responds to gentle stroking, a moving toy, or handclapping (able to slightly lift the head, tail, or a limb)	3
Lateral recumbency, does not respond to gentle stroking, a moving toy, or handclapping	4
